# Activated Charcoal-Alginate Platform for Simultaneous Local Delivery of Bioactive Agents: At the Nexus of Antimicrobial and Cytotoxic Activity of Zn^2+^ Ions

**DOI:** 10.3390/gels10110724

**Published:** 2024-11-08

**Authors:** Andrea Osmokrovic, Ivan Jancic, Zeljko Zizak, Marina Milenkovic, Bojana Obradovic

**Affiliations:** 1 University of Belgrade, Faculty of Technology and Metallurgy, Karnegijeva 4, 11000 Belgrade, Serbia; 2 University of Belgrade, Faculty of Pharmacy, Vojvode Stepe 450, 11000 Belgrade, Serbia; 3 Institute for Oncology and Radiology of Serbia, Pasterova 14, 11000 Belgrade, Serbia

**Keywords:** antimicrobial resistance, zinc ions, povidone–iodine, malignant wounds, antifungal activity

## Abstract

Antimicrobial resistance (AMR) is a global public health threat that affects cancer patients more than the general population. In this work, a composite system based on Zn-alginate hydrogel and activated charcoal (AC) particles that, upon contact with physiological fluids, simultaneously releases bioactive agents (Zn^2+^ and AC particles impregnated with povidone–iodine) was designed to locally address specific problems characteristic for malignant wounds (MWs). This composite was comprehensively investigated in vitro regarding its morphology (field-emission scanning electron microscopy), Zn^2+^ release (flame atomic absorption spectrometry), iodine adsorption and desorption from AC particles (energy dispersive X-ray analysis and UV–visible spectroscopy) as well as its antimicrobial and antitumor activity. With respect to the ongoing AMR crises, antimicrobial activity was tested against a wide range of wild multi-drug resistant bacterial and yeast strains, all isolated from patient wounds. Since Zn^2+^ ions proved to be selectors of resistant strains of bacteria, the synergistic activity of AC particles and adsorbed iodine was shown to be crucial for excellent antibacterial activity. On the other hand, the synergy of AC particles and Zn^2+^ ions showed an equally strong antifungal effect. In addition, antimicrobial concentrations of Zn^2+^ ions showed cytotoxic activity against two cancer cell lines derived from cancers affecting skin either as metastatic cancer (breast cancer MDA-MB-453 cell line) or primary cancer of the skin (malignant melanoma Fem-X cell line), which enables Zn^2+^ ions to be further investigated as potent local agents targeting malignant cells.

## 1. Introduction

Antimicrobial resistance (AMR) occurs when microorganisms acquire genetic mutations over time and develop resistance against drugs used to eliminate them. The World Health Organization (WHO) declared AMR one of the top ten global public health threats that humanity is facing today. Overuse and/or misuse of antimicrobials in human and veterinary medicine is primarily responsible for AMR development. Drug-resistant microbes make existing antimicrobial treatments ineffective, thus jeopardizing all medical procedures across therapeutic areas. However, cancer patients are disproportionally affected by AMR since various cancer therapies, including surgery, radiotherapy, and chemotherapy, suppress the immune system of the patient and increase their susceptibility to infections. Concretely, one in five cancer patients undergoing treatments are hospitalized due to infections (e.g., bacterial pneumonia, sepsis, etc.), which are the second leading cause of death in cancer patients. In addition, patients with cancer have a three times greater risk of dying from a fatal infection than patients without cancer [1].

Malignant wounds (MWs) are skin lesions, caused by the infiltration or damage of the skin by malignant tumors. The prevalence of MWs in cancer patients is between 5 and 10%, and are most likely to happen in breast cancer, head and neck cancer, or malignant melanoma patients [2,3]. Breast cancer (BC) is one of the most common cancers among women, while malignant melanoma (MM) is responsible for a large majority of skin cancer-related deaths (75.2%) [2]. Life expectancy in people with MWs is limited, estimated to be not more than 6 to 12 months. Thus, the goal of care of MWs shifts from healing to a palliative approach, with the aim to relieve and improve the quality of life of the patient. MWs are accompanied by disturbing symptoms such as exudate, necrosis, infection, malodor, bleeding, and pain, which are all very difficult to cope with. Despite the limited data, an association between the severity of symptoms and the cutaneous microbiome of malignant wounds has been established e.g., bacterial growth greater than 100,000/g of tissue is linked with increased exudate [4] and, in combination with necrotic tissue, results in noxious odors, which even in moderate cases, can cause distress, embarrassment, and withdrawal from social contact of the patient. Therefore, wound dressings aimed for the local treatment of MWs mostly comprise an activated charcoal (AC) fabric in-between other layers, whose function is, due to its microporous structure, to adsorb small volatile molecules responsible for malodor.

However, the management of exudate, as well as bacterial and/or fungal infection control are equally important. Hydrogels, like alginate, are widely used due to their high sorption capacity since MWs can sometimes produce over a liter of exudate per day [5]. As the hydrogel absorbs wound exudate, cross-linking cations (such as calcium and zinc) are exchanged with Na^+^ and are released into the wound area, acting as biologically active agents. The biological functions of Ca^2+^ ions have been well known for a long time, while the important role of zinc in biological systems has been recognized more recently, designating zinc as “the calcium” of the twenty-first century [6]. Zinc is present in all forms of life and is an essential element for humans, since deficiency of such can lead to the development of different diseases, including cancer. On the other hand, high concentrations of zinc are toxic to the cells due to intracellular zinc overload and consequent cellular and mitochondrial stress [7]. In vitro, zinc has demonstrated efficacy in inducing apoptosis in glioma [8], bladder cancer [9], prostate cancer [10], human melanoma [11] as well as breast cancer cells [12,13]. In addition, metal ions including Zn^2+^ are known to have strong inhibitory activity against various microbial strains [14]. However, today, antimicrobial activity of any agent should be examined with consideration to the ongoing “silent pandemic” or AMR, so this subject needs further investigation. Along with the gelating ion release, alginate hydrogels may also be used for the encapsulation and controlled release of various bioactive molecules (e.g., small and large drug molecules, proteins, growth factors, hormones, enzymes, etc.) when exposed to biological fluids, which normally contain NaCl. The loading of therapeutics within the alginate-based system is an efficient way of sustaining their delivery to the wound site for better therapeutic efficacy and local bioavailability. This can be beneficial in wounds with heavy exudate like MWs, in which drugs like, e.g., antimicrobials are rapidly being inactivated after local application, so other ways of their delivery should be considered.

Previously, we have successfully encapsulated particles of AC within the Ca- and Zn-alginate matrices, with the aim of in situ adsorption of not only small volatile molecules responsible for odor, but also larger molecules, wound products, and even microbial cells [15,16] due to mezzo- and macroporous structure of AC. We have also shown that AC particles can present very efficient carriers of other bioactive substances directly into the wound area, such as the highly potent antiseptic povidone–iodine (PVP-I) [15] or acyclovir as it was shown in the literature [17]. The previously-devised composite system based on Ca-alginate with the incorporated AC, impregnated with PVP-I, exhibited excellent antibacterial activity [15]. On the other hand, activity against fungi, precisely yeast *Candida* spp., was not as equally efficient. However, MWs are often colonized with yeasts because of prolonged antibiotic therapy, which implies that the antibacterial as well as the antifungal activity of the envisioned wound dressing is equally important.

In this work, we hypothesized that Zn^2+^ ions could contribute to achieving better antifungal activity of the alginate-AC composite system while at the same time maintaining excellent antibacterial activity against a wide range of multi-resistant bacteria. Furthermore, we aimed to examine if antimicrobial concentrations of released Zn^2+^ ions could induce cytotoxic effects against cancer cell lines derived from cancers affecting the skin, that is BC (cancer with high rates of metastasis to skin sites) or MM (primary skin cancer) and be considered as potential local healing agents. As a control, we have also investigated the resistance of normal tissue, such as fibroblasts and immune cells, present in wounds against Zn^2+^ ions.

## 2. Results and Discussion

### 2.1. Characterization of the Composite Beads

Composite ZnA/AC beads were successfully produced via extrusion of the AC suspension (20% *w*/*w*) in Na-alginate solution (0.5% *w*/*w*) into a gelling bath that contained Zn^2+^ ions, while the control ZnA beads were produced via extrusion of the Na-alginate solution (0.5% *w*/*w*) in the same manner. Upon drying, the composite beads practically retained the spherical shape and size (3.9 ± 0.2 and 3.4 ± 0.2 mm diameter of wet and dried beads, respectively; Figure S1, Supplementary Material).

The composite ZnA/AC/PVP-I beads were produced by overnight immersion of wet ZnA/AC beads in 10% *w*/*w* aqueous solution of PVP-I. The successful adsorption of PVP-I within the beads was indicated by de-colorization of the solution, which was confirmed by UV–vis spectroscopy, showing that the PVP-I concentration in the supernatant was below the detection level. Upon drying, these beads also retained an approximately spherical shape (3.4 ± 0.1 mm in diameter). An EDX analysis of ZnA/AC/PVP-I bead cross-sections has undoubtedly confirmed the presence of iodine in the beads (Figure S2, Supplementary Material).

The FE-SEM analyses of the ZnA/AC/PVP-I composite beads (Figure S3, Supplementary Material) showed rough morphology with discernible AC particles, visually very similar to the morphology of theZnA/AC composite beads, and at the same time different from the uniform morphology of the alginate hydrogel.

Desorption of PVP-I in physiological saline solution (0.9% *w*/*w* NaCl), used as a model for biological fluids, in which ZnA/AC/PVP-I beads were kept for 48 h at 37 °C under constant mixing was below the detection level as indicated by UV–vis analysis of the solution (Figure S4a, Supplementary Material). In addition, the dissolved ZnA/AC/PVP-I beads, after removal of the AC particles, exhibited a similar UV–vis spectrum without a visible peak at 351 nm (Figure S4b, Supplementary Material). These results confirmed that under physiologically relevant conditions, PVP-I is firmly adsorbed on AC particles and that its release in the surrounding medium (alginate or saline solution) is negligible.

### 2.2. Antimicrobial Activity Studies

#### 2.2.1. Antimicrobial Activity

**Figure 1:**
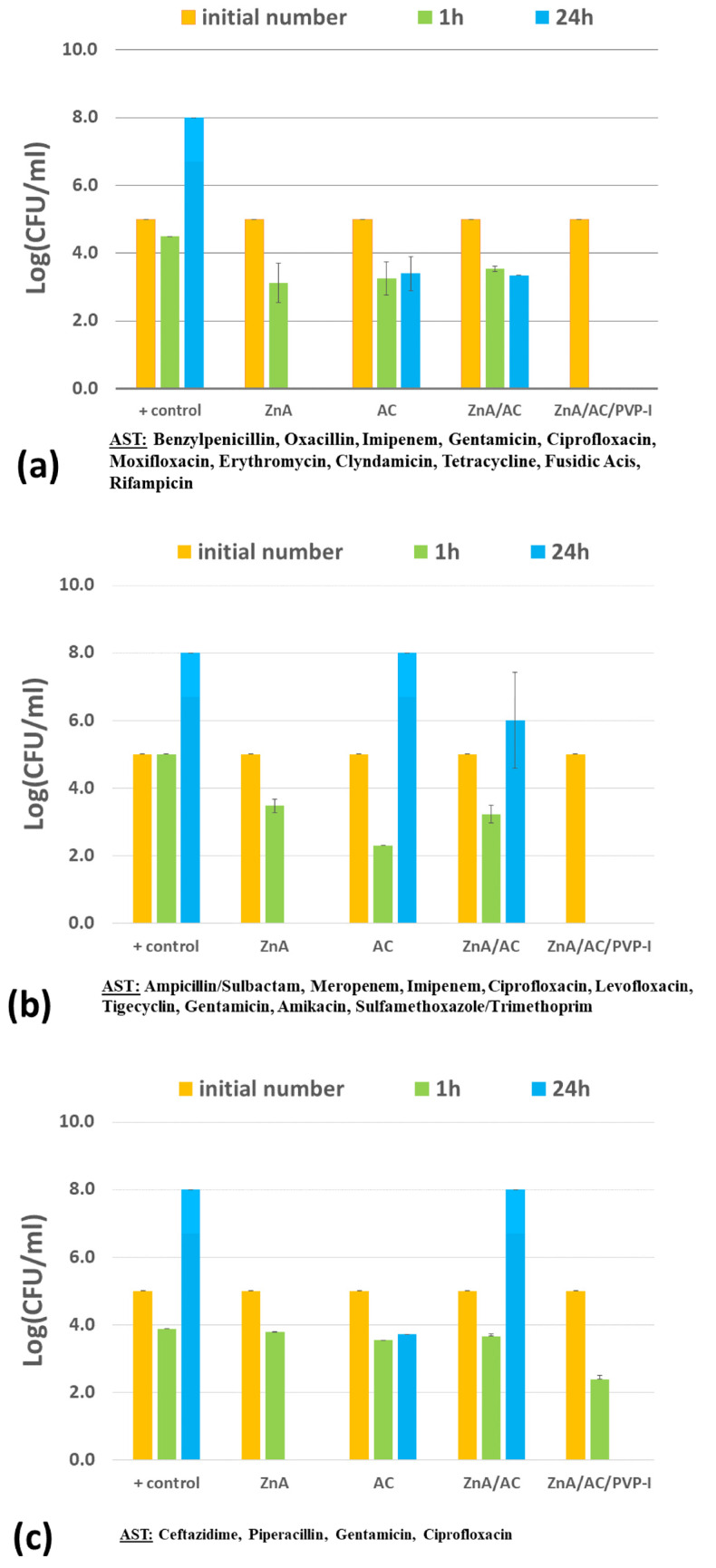

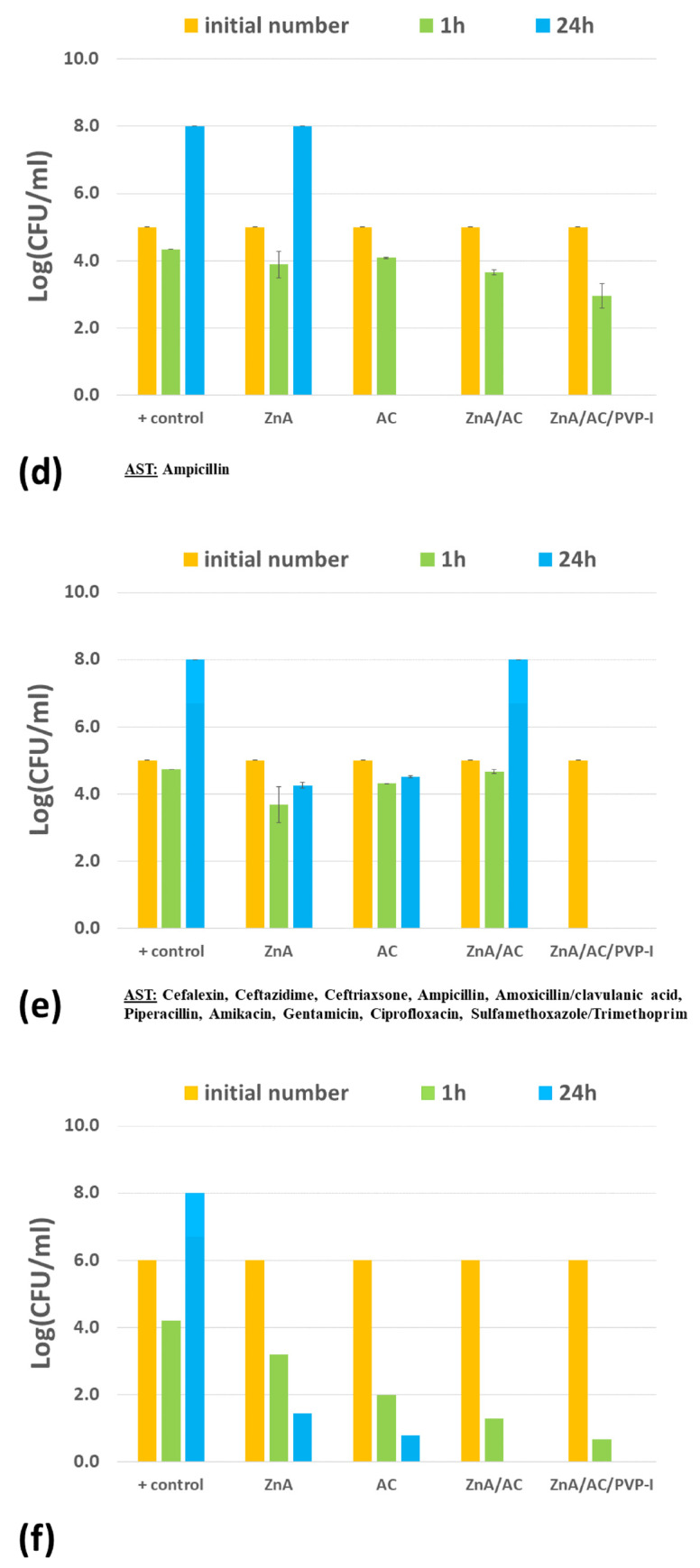
Antimicrobial activity of ZnA/AC and ZnA/AC/PVP-I beads against (**a**) MRSA; (**b**) *A. baumannii*; (**c**) *P. aeruginosa*; (**d**) *E. faecalis*; (**e**) *P. mirabilis*; (**f**) *Candida albicans*; controls were microbial suspensions without additives (+control), with AC powder and with ZnA beads; results are expressed as log(CFU/mL) and are average of *n* = 2 for bacterial strains while for the yeast *Candida albicans* are average of *n* = 12.

**Figure 2:**
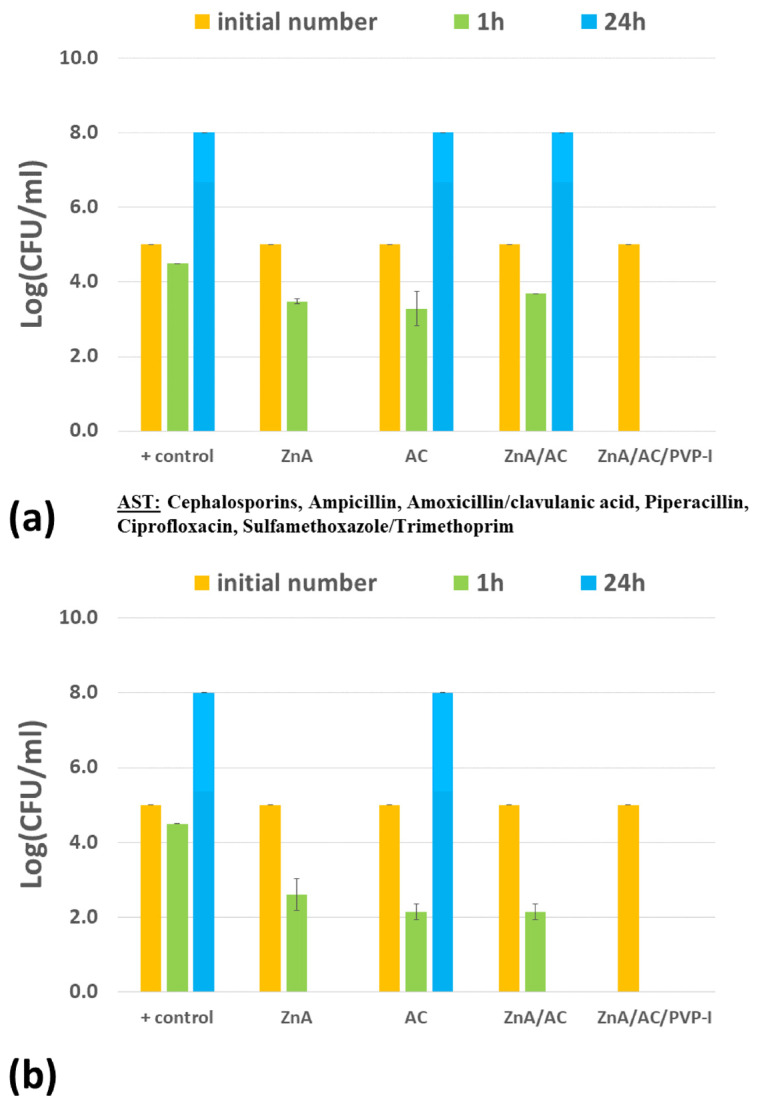
Antimicrobial activity of ZnA/AC and ZnA/AC/PVP-I beads against (**a**) ESBL-*E. coli*; (**b**) *E. coli ATCC 25922* (data for ZnA/AC, ZnA and AC are reprinted with permission from [16]); controls were microbial suspensions without additives (+control), with AC powder and with ZnA beads; results are expressed as log(CFU/mL) and are average of *n* = 2.

The antimicrobial activity of ZnA/AC and ZnA/AC/PVP-I beads was investigated in suspensions of 12 wild multidrug-resistant microbial strains isolated from patient wounds, including two Gram-positive bacterial strains (MRSA and *E. faecalis*), four Gram-negative bacterial strains (*A. baumannii*, *E. coli*, *P. mirabilis* and *P. aeruginosa*), and six isolates of *C. albicans* strain, while microbial suspensions, AC powder, and ZnA beads were used as controls (Figure 1 and Figure 2a). The ZnA/AC/PVP-I beads were tested in one additional suspension with the standard strain of *E. coli* ATCC 25922 (Figure 2b).

The experimental results show that composite beads impregnated with PVP-I exhibited excellent antibacterial activity, as expected. Precisely, the antibacterial activity was achieved in all examined bacterial suspensions. Additionally, in suspensions of MRSA, *A. baumannii*, *P. mirabilis*, *E. coli* and *E. coli* ATCC 25922 the bacteria were completely eliminated after only 1 h of contact (Figure 1a,b,e and Figure 2). On the other hand, the composite ZnA/AC beads achieved bacteriostatic activity in suspensions of MRSA and *A. baumannii* while in the case of *E. faecalis*, the bacteria were completely eliminated from the suspension (Figure 1a,b,d). Zn^2+^ ions alone, released from control ZnA beads, completely eliminated bacteria from suspensions of MRSA, *A. baumannii*, *P. aeruginosa* and *E. coli* (Figure 1a–c and Figure 2a), bacteriostatic effect was achieved in the suspension of *P. mirabilis* (Figure 1e)*,* while the effects were not noticed in the suspension of *E. faecalis* (Figure 1d).

In the suspension of the yeast *C. albicans*, the composite beads, with and without PVP-I (ZnA/AC/PVP-I and ZnA/AC, respectively), completely eliminated yeast cells from the suspension after a 24 h period, while the controls (AC and ZnA beads), during the same time period, significantly reduced the number of cells but did not achieve the complete fungicidal effect (Figure 1f).

#### 2.2.2. Zn^2+^ Release

**Figure 3:**
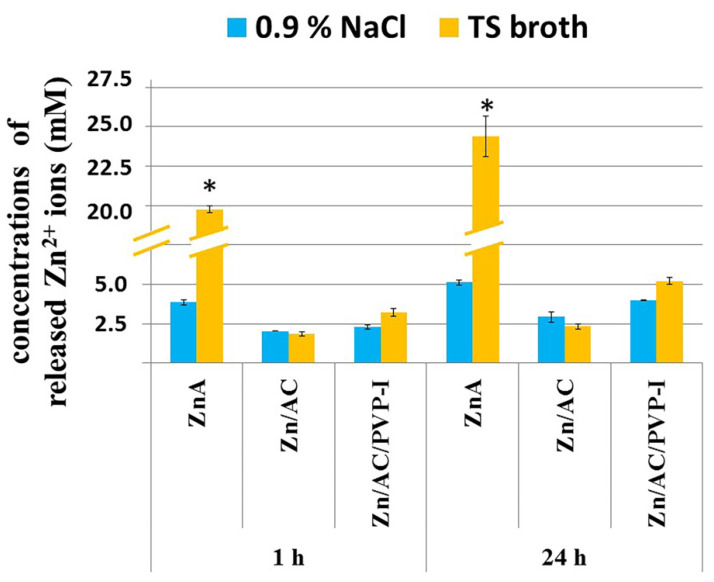
Concentrations of released Zn^2+^ ions from ZnA, ZnA/AC and ZnA/AC/PVP-I beads measured in physiological saline solution (0.9% *w*/*w* NaCl, pH 5.5) and TS broth (pH 7.3) after the periods of 1 h and 24 h (results are average of *n* = 3). Asterisks (*) indicate statistically significant differences (*p* < 0.05).

In order to elucidate the effects of Zn^2+^, the released concentrations from the Zn-alginate-based beads (ZnA, ZnA/AC and ZnA/AC/PVP-I) were measured in studies parallel to antimicrobial activity studies in TS broth (pH 7.3) and physiological saline solution (0.9% *w*/*w* NaCl, pH 5.5). The obtained results in both media were in the range from 2 to 5 mM, with the exception of approximately 5-fold higher values obtained for the ZnA beads in TS broth for both experimental time points (19.8 and 24.4 mM after 1 and 24 h, respectively, Figure 3).

### 2.3. Cytotoxicity Studies

**Figure 4:**
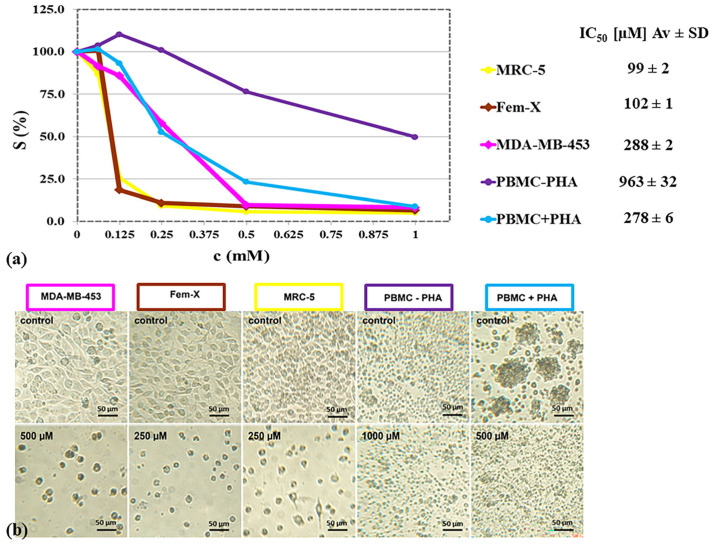
(**a**) Survival of malignant and normal cell lines and PBMC (without or with PHA) as determined by the MTT, after 72 h of continuous action of applied concentrations of ZnCl_2_ (SDs < 3%) and concentrations of ZnCl_2_ which induced 50% decrease (IC_50_) in studied cell lines and PBMC; (**b**) Optical micrographs of studied cell lines and PBMC after cultivation for 72 h in medium alone (control, upper row), or in medium with added ZnCl_2_ (concentrations are shown in the micrographs and correspond approximately to 2 × IC_50_), scale bar = 50 µm).

The antiproliferative activity of Zn^2+^ ions was tested against two tumor cell lines, MDA-MB-453 and Fem-X, as well as against a non-cancerous cell line, MRC-5, and against PBMC non-stimulated and stimulated with PHA. Zn^2+^ ions exerted a dose-dependent antiproliferative action toward investigated cell lines and PBMC, detected by the MTT (Figure 4). Melanoma Fem-X and normal MRC-5 cell lines have shown the most susceptibility to Zn^2+^ ions, breast carcinoma MDA-MB-453 cells and PHA-stimulated PBMC have exerted higher tolerance while non-stimulated PBMC have shown to be the most resistant to Zn^2+^ ions.

### 2.4. Discussion

In this study, a composite system based on Zn-alginate hydrogel and AC particles with an adsorbed bioactive substance (i.e., PVP-I used in the present work) was produced in the form of beads and investigated with the aim to be particularly suited for the treatment of MWs. It should be added that this composite could be produced in other forms, such as fibers, films, and sheets. In this potential wound dressing, each component (alginate, Zn^2+^ ions, AC particles and PVP-I) is intended to have an additional and complementary function, and to address a specific problem in a MW. In the present approach, alginate hydrogels provide swelling and therefore absorption of large amounts of exudates often present in this type of wounds, while, at the same time, they enable the continuous and efficient release of two active components, Zn^2+^ ions and AC particles, directly into the wound area. Thus, their local delivery is enabled for a prolonged period of time, while potential unwanted systemic effects are being minimized. The simultaneous application of compatible bioactive agents is often used to treat resistant pathogens, but it is also used in the development of novel envisioned antimicrobial drugs, such as complementary supramolecular drugs associates [18]. Furthermore, local delivery systems are especially attractive for cancer treatment applications because chemotherapeutics have poor bioavailability at the tumor site which consequently leads to higher drug dosages and enhanced toxicity. Zn^2+^ ions, as a first active component, act as an antimicrobial and cytotoxic agent. AC particles, as a second active component, are intended for in situ adsorption of malodor molecules, toxins, degradation products and microorganisms, as well as for the delivery of other active substances, in this case PVP-I, an antiseptic for which, to date, instances of acquired or cross-drug resistance have not been documented. It should be noted that PVP-I was efficiently adsorbed onto the AC particles within ZnA/AC beads without any significant release, neither into the surrounding alginate matrix nor in the physiological saline solution over 48 h. This is very important with regards to preventing often reported systemic iodine absorption after prolonged medical usage of PVP-I [19,20].

In order to elucidate the effects of each individual component in the composite system, antimicrobial studies were performed with several controls (i.e., AC powder and ZnA beads in addition to microbial suspensions alone), while parallel studies were performed to determine released Zn^2+^ concentrations. Since alginate is a pH sensitive hydrogel with higher swelling degrees and faster drug release in alkaline environments as compared to acidic conditions [21], release of Zn^2+^ ions from composite ZnA/AC and ZnA/AC/PVP-I beads as well as from control ZnA beads was measured in two types of media, saline solution (pH 5.5) and TS broth (pH 7.3). It should be noted that pH of wound exudates in chronic wounds is in the range of 7.15–8.9 [22]. As expected, the control ZnA beads, at both investigated time points released much higher amounts of Zn^2+^ ions in TS broth, yielding approximately 5-fold higher concentrations, than in the saline solution. On the other hand, the composite beads (ZnA/AC and ZnA/AC/PVP-I) released similar concentrations of Zn^2+^ ions in both media. We have previously shown that the AC type applied in the present study and aimed for medical use did not exhibit affinity towards Zn^2+^ ions [16] but it interacted with alginate chains and prevented expansion of the gel network by alginate adsorption [15]. Thus, incorporated AC particles indirectly influenced the concentration of released Zn^2+^ ions into the surrounding media by reducing the impact of pH on the alginate hydrogel. This leads to more predictable ion release kinetics from the alginate matrix.

Even though control ZnA beads by far released the highest concentrations of Zn^2+^ ions in TS broth (Figure 3), the best antibacterial result was achieved by composite beads impregnated with PVP-I (ZnA/AC/PVP-I) due to additional actions of AC particles efficiently adsorbing bacterial cells followed by bactericidal effects of PVP-I as it was shown previously for the Ca-alginate system [15]. In the present study, antimicrobial effects were investigated against 12 clinical isolates of wild multidrug-resistant bacterial and yeast strains. It should be noted that several of these bacterial strains are listed by the WHO in critical priority category or priority 1 (carbapenem—resistant *A. baumannii*, carbapenem—resistant *Enterobacteriaceae* and ESBL—producing *E. coli*) and high priority category or priority 2 (methicillin-resistant *Staphylococcus aureus*). The effects of released Zn^2+^ ions could be clearly perceived by inspecting the cultures with ZnA beads showing that after 24 h bactericidal effects were found in suspensions of MRSA, *A. baumannii*, *E. coli* and *P. aeruginosa*, bacteriostatic effect was found in the suspension of *P. mirabilis* and a certain antifungal effect was found in the suspension of *C. albicans*, while the effect could not be noticed in the suspension of *E. faecalis* (Figure 1). It is interesting to note that although the released Zn^2+^ concentrations after 1 and 24 h were similar (19.8 mM vs. 24.4 mM, Figure 3) the effects were observed in most cases only at the second time point. These results indicate that a certain period of time is needed for released Zn^2+^ ions to enter the cell and achieve antimicrobial effect. The obtained results are also in agreement with literature data reporting that MRSA and *A. baumannii* are very susceptible to Zn^2+^ ions [23,24]. Accordingly, in the suspensions of these two bacteria, the negative effect on the bacterial growth was achieved by all three types of beads with the weakest effects of ZnA/AC beads releasing the lowest Zn^2+^ concentration (~2.3 mM after 24 h). Indeed, MIC values for these bacteria were reported to be in the range of 0.25–2 mM Zn^2+^ [23,24].

Experimental results obtained in the present study suggest that *E. coli* is more tolerable to Zn^2+^ ions since composite ZnA/AC beads did not induce negative effect against wild multidrug-resistant strain ESBL-*E. coli* (Figure 2a). However, our previous study showed that, under the same experimental settings, standard strain of *E. coli* (ATCC 25922) was very susceptible to Zn^2+^ ions since it was completely eliminated from the suspension after 24 h by the same composite ZnA/AC beads (Figure 2b). Literature data show that higher tolerance to Zn^2+^ ions can be connected to the resistance to certain antimicrobials (e.g., sulphametoxasol, ciprofloxacin) or to acquired multidrug resistance [25]. This means that Zn^2+^ ions can act as a selector of resistant strains, which was confirmed by the results obtained in the present study as well. This finding could also be of relevance in everyday life, since Zn compounds are widely used in ointments and skin preparations aimed to be used from a very early age, which could potentially influence the skin flora [23].

On the other hand, *P. aeruginosa*, *E. faecalis* and *P. mirabilis* are bacterial strains, which were shown not to be very susceptible to Zn^2+^ ions with the latter one being the most resistant even at the highest concentration of ~24.4 mM Zn^2+^ released by the control ZnA beads after 24 h (Figure 1c–e). These results are in accordance with literature data, which show that *P. aeruginosa*, *Proteus* sp. and *Enterococcus* sp. belong to bacteria which possess higher natural tolerance to Zn^2+^ ions with MIC values between 8 and 32 mM [26,27]. Also, it should be noted that the resistance to antibiotic ampicillin can be connected to the higher Zn^2+^ tolerance. Specifically, it has been shown that 50% of the isolates resistant to ampicillin are resistant to Zn^2+^ ions as well [28]. Since the two wild isolates of *P. mirabilis* and *E. faecalis* are resistant to ampicillin (Table 1) this could also explain tolerance of these strains to such high Zn^2+^ concentrations. However, AC was very efficient against *E. faecalis* bacteria which were completely eliminated from the suspensions by ZnA/AC and AC particles (Figure 1d), most probably by adsorption. The same results were obtained in our previous study with composite beads based on Ca-alginate and AC [15].

As noticed above, Zn^2+^ released from the ZnA beads exhibited some antifungal effects corresponding to the literature data, indicating that *Candida* spp. is not very susceptible to metal ions such as Zn^2+^ [29]. Also, it is interesting to note that in our previous studies, under the same experimental settings, the composite beads based on Ca-alginate and incorporated AC particles with adsorbed PVP-I during the 24 h period, have achieved only fungistatic effect [15], which indicates that AC with adsorbed iodine is also not sufficiently efficient against the yeast *C. albicans*. Thus, the observed strong antifungal effect of both types of composite beads (ZnA/AC and ZnA/AC/PVP-I) in the present study could be attributed to a synergistic effect of the AC particles and released Zn^2+^ ions.

In order to investigate potential antiproliferative activity of the developed composite systems, we have studied the effects of Zn^2+^ ions on a BC cell line as a model for cancer with high rates of metastasis to skin sites and a MM cell line as a model of primary skin cancer, while normal fibroblasts and immune cells served as controls as cells present in the wound. All investigated cell types have shown an exerted dose—dependence of their susceptibility to Zn^2+^ ions, with non-stimulated PBMC’s exhibiting the highest tolerance. This result is expected since cells that are not replicating are more resistant to external stressors than the replicating ones [30]. Breast carcinoma MDA-MB-453 cells have been shown to be more tolerable to Zn^2+^ ions than the melanoma Fem-X cells. According to the literature, BC tissues show the increased expression of numerous zinc importers, which leads to a hyper-accumulation of this metal in the cells, with respect to the normal tissue, as well as in metastatic breast tissues in comparison to a non-metastatic one [31]. For most types of normal cells, the total cellular zinc concentration is in the range 0.1–0.5 mM [32], while in invasive cancer tissue obtained after mastectomy, it can be as high as 1.9 mM (corresponds to 126 ppm [33]). However, recent evidence shows that zinc in breast cancer cells is compartmentalized, which protects the cells from the cytotoxic effects of high cytosolic levels of zinc [34]. So, even if zinc is hyper-accumulated in malignancy, the effect of elevated cytoplasmic zinc is cytotoxic. Furthermore, patients with advanced BC have lower serum zinc levels in comparison to patients with early BC and benign breast diseases (88.6 µg/dL vs. 115.1 µg/dL, *p* < 0.001) which suggests that Zn^2+^ ions are imported from the serum into the cytosol by invasive breast tumor cells [35]. Many studies have been conducted regarding the effectiveness of using zinc either orally or intravenously as a complementary treatment in cancer patients, but to date, conclusive results regarding its benefits are lacking [36]. However, it has been reported that multiple local administrations of 3 mM Zn^2+^ ions directly into the malignant wound area resulted in a profound negative effect on the growth of human prostate tumor xenografts [37]. This zinc treatment approach may be applicable in hepatocellular, pancreatic, and ovarian malignant cells as well [38]. So, in order to induce toxic effects, cancer cells have to be exposed to increased levels of zinc. Likewise, direct prolonged exposure of cancerous tissue in MWs at supra-physiological concentrations of released Zn^2+^ ions could have a positive effect due to the zinc-induced cell death while an efficient zinc treatment regimen would require a zinc-delivery system or mechanism that would facilitate an uptake and accumulation of zinc into the malignant cells [38]. In addition, locally available Zn^2+^ ions could reduce the ion import from serums and thus positively influence the serum Zn levels important for normal functions of other cells and organs and especially the immune system of the patient.

## 3. Conclusions

In this work, we have successfully produced a composite system based on Zn-alginate hydrogel and AC particles with adsorbed PVP-I as a model bioactive substance especially designed to address in situ specific symptoms of MWs. The necrotic tissue present in MWs is an ideal breeding ground for various microbes which are further responsible for intensive malodor; therefore, a wound dressing designed for MW application should possess strong antibacterial and antifungal activity. Specifically, the synergy of AC particles and adsorbed iodine enabled fast and efficient antibacterial activity against a wide range of wild multidrug-resistant strains without the danger of a systemic absorption of iodine, while the synergy of AC particles and Zn^2+^ ions enabled an equally strong antifungal effect. Furthermore, our study clearly showed that Zn^2+^ ions are selectors of resistant strains of bacteria, and, as such, are not suitable to be the main and/or the only bearers of antimicrobial activity. This is very relevant with regards to the ongoing global AMR crises, since new antimicrobial substances are intensively searched for, and metal ions are seen as one of the promising alternatives. Furthermore, released concentrations of Zn^2+^ ions have been shown to be cytotoxic against both investigated cancer cell lines in monolayers derived from cancers affecting skin which opens the possibility of their usage as local therapeutic agents. However, further experiments in 3D cultures are needed in order to precisely understand under which circumstances specific cancer cells could be targeted by zinc ions. On the other hand, since antimicrobial concentrations of Zn^2+^ ions would be in contact with normal fibroblasts as well and since they are selectors of resistant strains, their usage as antimicrobial agents aimed for non-malignant wounds should be avoided. To sum up, complementary and synergistic activity of the initial low-cost components of the composite ZnA/AC/PVP-I beads resulted in very pronounced antimicrobial and antitumor effects delegating this system to be especially attractive for possible MW dressing applications.

## 4. Materials and Methods

### 4.1. Preparation of Alginate-Based Beads

#### 4.1.1. Preparation of Zinc Alginate Composite Beads (ZnA/AC)

Zinc alginate beads with incorporated activated charcoal particles (ZnA/AC) were prepared as previously described [16]. Briefly, fine AC powder (MEKS 95, Trayal, Kruševac, Serbia) was dispersed in aqueous solution of sodium alginate (A2033, medium viscosity, Sigma, St. Louis, MO, USA) by using the mechanical stirrer Ultra-Turrax^®^ T25 (Janke and Kunkel Ika-Labortechnik, Staufen, Germany) at 20,000 rpm for 5 min. The obtained suspension, with final concentrations of 20% *w*/*w* AC and 0.5% *w*/*w* alginate, was manually extruded through a blunt edge stainless steel needle (16G) into a gelling bath (1.8% *w*/*w* Zn[NO_3_]_2_·6H_2_O, Sigma, St. Louis, MO, USA). The beads were left in the bath for additional 30 min to complete gelling and then washed in distilled water for several times. The obtained ZnA/AC beads were either used for impregnation with PVP-I (Hemofarm AD, Vrsac, Serbia) or air-dried sterile in laminar hood at room temperature until the constant weight.

The control zinc alginate (ZnA) beads were prepared in the same manner by using the same aqueous Na-alginate solution (0.5% *w*/*w*), which was then manually extruded into the same gelling bath (1.8% *w*/*w* Zn[NO_3_]_2_·6H_2_O). The beads were left for 30 min in the bath, washed in distilled water several times, and air dried at room temperature in laminar hood until the constant weight.

#### 4.1.2. Preparation of Zinc Alginate Composite Beads Impregnated with PVP-I (ZnA/AC/PVP-I)

Zinc alginate beads with incorporated activated charcoal particles impregnated with PVP-I (ZnA/AC/PVP-I) were produced sterile, in a laminar hood, as described previously [15]. Briefly, 4 g of wet ZnA/AC beads were immersed overnight in 4 mL of a 10% *w*/*w* PVP-I aqueous solution, and then washed in distilled water several times. The beads were then air-dried under sterile conditions in laminar hood at room temperature until the constant weight.

In order to investigate the possible desorption of PVP-I under physiological conditions, dry ZnA/AC/PVP-I beads (corresponding to 2 g of wet weight) were placed in 20 mL of a physiological saline solution (0.9% *w*/*w* NaCl, Centrohem, Stara Pazova, Serbia) in closed 100 mL Erlenmeyer flasks, placed in a shaking water bath at 37 °C at 120 rpm. After 48 h the beads were separated from the medium and dissolved in 2% *w*/*w* sodium citrate (Himedia, Mumbai, Maharashtra, India). In order to remove the AC particles, the dissolved beads and the medium were each centrifuged at 600× *g* for 10 min and the presence of PVP-I in the supernatants was examined via UV-Vis spectroscopy (at least three measurements were carried out for each determination). The experiments were conducted in duplicates.

### 4.2. Characterization of the Composite Beads

#### 4.2.1. UV-Vis Spectroscopy

The presence of PVP-I in the solution samples was examined by UV–visible spectroscopy (UV-3100 spectrophotometer, MAPADA Instruments, Shanghai, China) at the wavelength of 351 nm, chosen according to the literature [39].

#### 4.2.2. Optical Microscopy and Bead Diameter Measurements

Optical microscopy images were acquired using a microscope Olympus 315 CX41RF (Olympus, Tokyo, Japan), supplied with a camera HDR-CX210 (Sony, Kanagawa, Tokyo, Japan). Beads were photographed in a Petri dish, supplied with a millimeter grid and the average bead diameter was calculated from measurements of at least 20 beads by using the image analysis program CellA316 (Olympus, Tokyo, Japan).

#### 4.2.3. Field—Emission Scanning Electron Microscopy [FE-SEM] and Energy Dispersive X-Ray (EDX) Analysis

The randomly-selected, dry ZnA/AC and ZnA/AC/PVP-I beads, were cross-sectioned in half using a scalpel. The morphology of the gold-coated cross-sections of ZnA/AC/PVP-I bead was examined by a MIRA 3 XMU field emission scanning electron microscope (Tescan USA Inc., Cranberry Twp, PA, USA), while an elemental analysis was performed on the ZnA/AC and ZnA/AC/PVP-I beads by using a INCAx-act LN2-free Analytical Silicon Drift Detector of characteristic X-ray with PentaFET^®^ Precision and Aztec 4.3 software package (Oxford instruments, Abingdon, UK), connected to a TESCAN Mira3XMU.

### 4.3. Antimicrobial Activity Studies

#### 4.3.1. Antimicrobial Activity

The antimicrobial activity of composite beads was investigated in suspensions of six wild multidrug-resistant bacterial strains (*Acinetobacter baumannii*, *Enterococcus faecalis*, *Escherichia coli*, MRSA, *Proteus mirabilis* and *Pseudomonas aeruginosa*) and six isolates of the wild yeast strain (*Candida albicans*). All strains were isolated from patient wounds and supplied by the City Institute for Public Health (Belgrade, Serbia). The results of the antimicrobial susceptibility tests (AST) are shown in Table 1. Also, one standard strain *Escherichia coli* ATCC 25922 was used as well.

Active cultures were prepared by transferring a loopful of cells from the stock into tubes that contained 10 mL of physiological saline solution (0.9% *w*/*w* NaCl). The suspensions were adjusted to 0.5 McFarland standard turbidity (corresponding to 1.5 × 10^8^ colony forming units (CFU/mL)). The microbial cultures were further diluted with physiological saline solution to the final concentration of 10^7^ CFU/mL.

Dry ZnA/AC and ZnA/AC/PVP-I beads (corresponding to 4 g wet weight) were added to 100 mL Erlenmeyer flasks followed by an addition of 10 mL of sterile tryptic soy broth (TS, Himedia, Mumbai, Maharashtra, India). Aliquots of 0.1 mL microbial suspensions were inoculated in each flask so that the initial number in TS broth was approximately 10^5^ CFU/mL for bacteria and 10^6^ CFU/mL for yeast. The prepared samples were incubated at 37 °C for 24 h. Microbial cultures in TS broth without the beads served as a positive control, TC broth alone as a negative control, while cultures with AC powder (1.2 g corresponding to the AC amount in ZnA/AC beads added to flasks) and dry ZnA beads (corresponding to 4 g wet weight) were additional controls. After 1 h and 24 h of incubation, 1 mL liquid samples were withdrawn aseptically from each flask and centrifuged (Centrifuge 5804, Eppendorf, Mississauga, ON, Canada) at 600× *g* for 10 min in order to remove AC particles. Then, 0.1 mL of the supernatant was withdrawn and cultured on TS agar in a Petri dish. Agar plates were incubated at 37 °C for 24 h and numbers of microbial colonies were assessed to obtain the number of viable cells in suspension. All experiments were carried out in duplicates.

#### 4.3.2. Zn^2+^ Release

In a parallel study, concentrations of released Zn^2+^ ions from dry ZnA, ZnA/AC and ZnA/AC/PVP-I beads were measured after 1 h and 24 h of incubation in TS broth and in physiological saline solution (0.9% *w*/*w* NaCl) under the same conditions as in antimicrobial activity studies. Liquid samples (1 mL) were centrifuged at 5000× *g* for 5 min in order to remove AC particles from the solutions and Zn^2+^ concentrations were determined by flame atomic absorption spectrometry (Perkin Elmer 3100 spectrometer, Perkin Elmer, Waltham, MA, USA). All experiments were carried out in triplicates.

#### 4.3.3. Statistical Analysis

All values are expressed as mean ± standard deviation. Mean values were analyzed using one-way ANOVA. The Tukey post hoc test was performed for means comparison (Origin Pro 8 (1991–2007) computer package, Origin Lab Co., Northampton, MA, USA). Data were considered significantly different when *p* < 0.05.

### 4.4. Cytotoxicity Studies

#### 4.4.1. Preparation and Treatment of Cells

Two cancerous cell lines, human breast adenocarcinoma MDA-MB-453 and human melanoma Fem-X, and a non-cancerous cell line, human embryonic lung fibroblasts MRC-5 (all from ATCC, Manassas, VA, USA) were grown in complete nutrient medium (RPMI-1640 medium, 2 mM L-glutamine, 1% penicillin/streptomycin, all from Sigma, St. Louis, MO, USA) enriched with 10% fetal bovine serum (FBS, Biochrom AG, Berlin, Germany). Target adherent cells MDA-MB-453 (3000 cells/well), Fem-X (3000 cells/well) and MRC-5 (5000 cells/well) were seeded into the wells of a 96-well flat-bottom microtiter plate. After the cell adherence over 24 h, complete medium with five different working concentrations (0.062, 0.125, 0.25, 0.5 and 1 mM) of ZnCl_2_ (Sigma, USA) was added to the wells. In the controls only the complete medium was added. Culture medium with corresponding concentrations of ZnCl_2_, but without cells, was used as a blank.

Peripheral blood mononuclear cells (PBMC) were separated from whole heparinized blood samples (obtained from healthy volunteers) by Lymphoprep (Nycomed, Oslo, Norway) gradient centrifugation. Interface cells, washed three times with Haemaccel (aqueous solution supplemented with 145 mM Na^+^, 5.1 mM K^+^, 6.2 mM Ca^2+^, 145 Mm Cl^−^ and 35 g/L gelatin polymers, pH 7.4, Abbott, Chicago, IL, USA), were counted and re-suspended in complete nutrient medium. PBMC were seeded at the density of 150,000 cells per well in complete medium only, or in complete medium enriched with 5 μg/mL of phytohaemagglutinin (PHA, Welcome Diagnostics, Hartford, Essex, UK) in 96-well microtiter plates. Two hours later, five different concentrations of ZnCl_2_ were added to the wells with non-stimulated and PHA stimulated PBMCs in order to achieve desired concentrations of ZnCl_2_ (0.062, 0.125, 0.25, 0.5 and 1 mM).

Cells were observed before and after treatment under the bright-field inverted microscope and photographed with E-410 camera Olympus (Tokyo, Japan).

#### 4.4.2. Determination of Cell Survival

All cultures were incubated for 72 h, and the effects of ZnCl_2_ on the survival of cancer and normal cells were determined by using the microculturetetrazolium test (MTT), according to Mosmann [40], with a modification by Ohno and Abe [41], which is described in the Supplementary Material.

## Figures and Tables

**Table 1:** Results of AST of wild microbial strains used in the present work.

Bacteria Isolated from Wounds	Resistance Information
*Acinetobacter baumannii*	Ampicillin/Sulbactam, Meropenem, Imipenem, Ciprofloxacin, Levofloxacin, Tigecyclin, Gentamicin, Amikacin, Sulfamethoxazole/Trimethoprim
*Enterococcus faecalis*	Ampicillin
ESBL *Escherichia coli* (ESBL-EC *)	Cephalosporins, Ampicillin, Amoxicillin/clavulanic acid, Piperacillin, Ciprofloxacin, Sulfamethoxazole/Trimethoprim
Methicillin-resistant *Staphylococcus aureus* (MRSA) **	Benzylpenicillin, Oxacillin, Imipenem, Gentamicin, Ciprofloxacin, Moxifloxacin, Erythromycin, Clyndamicin, Tetracycline, Fusidic Acis, Rifampicin
*Proteus mirabilis*	Cefalexin, Ceftazidime, Ceftriaxsone, Ampicillin, Amoxicillin/clavulanic acid, Piperacillin, Amikacin, Gentamicin, Ciprofloxacin, Sulfamethoxazole/Trimethoprim
*Pseudomonas aeruginosa*	Ceftazidime, Piperacillin, Gentamicin, Ciprofloxacin

* extended-spectrum β-lactamase-producing *Escherichia coli*. ** mecA gene confirmed.

## Data Availability

The original contributions presented in the study are included in the article/Supplementary Material, further inquiries can be directed to the corresponding author.

## Supplementary Materials

The following supporting information can be downloaded at: https://www.mdpi.com/article/10.3390/gels10110724/s1, Figure S1: (a) wet ZnA/AC beads; (b) dry ZnA/AC beads (scale bar: 5 mm); Figure S2: EDX mapping of (a) ZnA/AC/PVP-I and (b) ZnA/AC composite beads; Figure S3: FE-SEM micrograph of cross-section of dry ZnA/AC/PVP-I bead (scale bar: 10 μm); Figure S4: UV-Vis spectra of the: (a) saline solution; (b) dissolved ZnA/AC/PVP-I beads after 48 h in physiological saline solution.

## References

[1] Nanayakkara A.K., Boucher H.W., Fowler V.G., Jezek A., Outterson K., Greenberg D.E. (2021). Antibiotic resistance in the patient with cancer: Escalating challenges and paths forward. CA Cancer J. Clin..

[2] Alexander S. (2009). Malignant fungating wounds: Epidemiology, aetiology, presentation and assessment. J. Wound Care.

[3] Gibson S., Green J. (2013). Review of patients’ experiences with fungating wounds and associated quality of life. J. Wound Care.

[4] Vardhan M., Flaminio Z., Sapru S., Tilley C.P., Fu M.R., Comfort C., Li X., Saxena D. (2019). The microbiome, malignant fungating wounds, and palliative care. Front. Cell. Infect. Microbiol..

[5] Alexander S. (2009). Malignant fungating wounds: Key symptoms and psychosocial issues. J. Wound Care.

[6] Plum L.M., Rink L., Haase H. (2010). The essential toxin: Impact of zinc on human health. Int. J. Environ. Res. Public Health.

[7] Knies K.A., Li Y.V. (2021). Zinc cytotoxicity induces mitochondrial morphology changes in HeLa cell line. Int. J. Physiol. Pathophysiol. Pharmacol..

[8] Wang Y., Zhang S., Li S.J. (2013). Zn^2+^ induces apoptosis in human highly metastatic SHG-44 glioma cells, through inhibiting activity of the voltage-gated proton channel Hv1. Biochem. Biophys. Res. Commun..

[9] Hong S.-H., Choi Y.S., Cho H.J., Lee J.Y., Hwang T.-K., Kim S.W. (2012). Induction of apoptosis of bladder cancer cells by zinc-citrate compound. Korean J. Urol..

[10] Hong S.H., Choi Y.S., Cho H.J., Lee J.Y., Kim J.C., Hwang T.K., Kim S.W. (2012). Antiproliferative effects of zinc-citrate compound on hormone refractory prostate cancer. Chin. J. Cancer Res..

[11] Provinciali M., Pierpaoli E., Bartozzi B., Bernardini G. (2015). Zinc induces apoptosis of human melanoma cells, increasing reactive oxygen species, p53 and FAS ligand. Anticancer Res..

[12] Provinciali M., Donnini A., Argentati K., DiStasio G., Bartozzi B., Bernardini G. (2002). Reactive oxygen species modulate Zn^2+^-induced apoptosis in cancer cells. Free Radic. Biol. Med..

[13] Lappano R., Malaguarnera R., Belfiore A., Maggiolini M. (2016). Recent advances on the stimulatory effects of metals in breast cancer. Mol. Cell. Endocrinol..

[14] Poole K. (2017). At the nexus of antibiotics and metals: The impact of Cu and Zn on antibiotic activity and resistance. Trends Microbiol..

[15] Osmokrovic A., Jancic I., Vunduk J., Petrovic P., Milenkovic M., Obradovic B. (2018). Achieving high antimicrobial activity: Composite alginate hydrogel beads releasing activated charcoal with an immobilized active agent. Carbohydr. Polym..

[16] Osmokrovic A., Jancic I., Jankovic-Castvan I., Petrovic P., Milenkovic M., Obradovic B. (2019). Novel composite zinc-alginate hydrogels with activated charcoal aimed for potential applications in multifunctional primary wound dressings. Chem. Ind..

[17] Yadavalli T., Ames J., Agelidis A., Suryawanshi R., Jaishankar D., Hopkins J., Thakkar N., Koujah L., Shukla D. (2019). Drug-encapsulated carbon (DECON): A novel platform for enhanced drug delivery. Sci. Adv..

[18] Sahoo P. (2024). Complementary supramolecular drug associates in perfecting the multi drug therapy against multi drug resistant bacteria. Front. Immunol..

[19] DelaCruz F., Harper Brown D., Leikin J.B., Franklin C., Hryhorczuk D.O. (1987). Iodine absorption after topical administration. West J. Med..

[20] Nesvadbova M., Crosera M., Maina G., Filon F.L. (2015). Povidone iodine skin absorption: An ex-vivo study. Toxicol. Lett..

[21] Chuang J.-J., Huang Y.-Y., Lo S.-H., Hsu T.-F., Huang W.-Y., Huang S.-L., Lin Y.-S. (2017). Effects of pH on the shape of alginate particles and its release behavior. Int. J. Polym. Sci..

[22] Gethin G. (2007). The significance of surface pH in chronic wounds. Wounds UK.

[23] Van Alen S., Kaspar U., Idelevich E.A., Kock R., Becker K. (2018). Increase of zinc resistance in German human derived livestock-associated MRSA between 2000 and 2014. Vet. Microbiol..

[24] Vaidya M., McBain A.J., Banks C.E., Whitehead K.A. (2019). Single and combined antimicrobial efficacies for nine metal ion solutions against *Klebsiella pneumoniae*, *Acinetobacter baumannii* and *Enterococcus faecium*. Int. Biodeterior. Biodegrad..

[25] Becerra-Castro C., Machado R.A., Vaz-Moreira I., Manaia C.M. (2015). Assessment of copper and zinc salts as selectors of antibiotic resistance in Gram-negative bacteria. Sci. Total Environ..

[26] Teitzel G.M., Parsek M.R. (2003). Heavy metal resistance of biofilm and planktonic *Pseudomonas aeruginosa*. Appl. Environ. Microbiol..

[27] Lansdown A.B.G., Mirastschijski U., Stubbs N., Scanlon E., Ågren M.S. (2007). Zinc in wound healing: Theoretical, experimental, and clinical aspects. Wound Repair Regen..

[28] Sabry S.A., Ghozlan H.A., Abou-Zeid D.M. (1997). Metal tolerance and antibiotic resistance patterns of a bacterial population isolated from sea water. J. App. Microbiol..

[29] Harrison J.J., Ceri H., Yerly J., Rabiei M., Hu Y., Martinuzzi R., Turner R.J. (2007). Metal ions may suppress or enhance cellular differentiation in *Candida albicans* and *Candida tropicalis* biofilms. Appl. Environ. Microbiol..

[30] Valeriote F., van Putten L. (1975). Proliferation-dependent cytotoxicity of anticancer agents: A review. Cancer Res..

[31] Kagara N., Tanaka N., Noguchi S., Hirano T. (2007). Zinc and its transporter ZIP10 are involved in invasive behavior of breast cancer cells. Cancer Sci..

[32] Eide D.J. (2006). Review: Zinc transporters and the cellular trafficking of zinc. Biochim. Biophys. Acta.

[33] Raju G.N., Sarita P., Kumar M.R., Murty G.R., Reddy B.S., Lakshminarayana S., Vijayan V., Lakshmi P.R., Gavarasana S., Reddy S.B. (2006). Trace elemental correlation study in malignant and normal breast tissue by PIXE technique. Nucl. Instrum. Methods Phys. Res. Sect. B.

[34] Lopez V., Foolad F., Kelleher S.L. (2011). Zn T2-over expression represses the cytotoxic effects of zinc hyper-accumulation in malignant metallothionei-null T47D breast tumor cells. Cancer Lett..

[35] Feng Y., Zenga J.W., Ma Q., Zhang S., Tang J., Feng J.F. (2020). Serum copper and zinc levels in breast cancer: A meta-analysis. J. Trace Elem. Med. Biol..

[36] Hoppe C., Kutschan S., Dörfler J., Büntzel J., Huebner J. (2021). Zinc as a complementary treatment for cancer patients: A systematic review. Clin. Exp. Med..

[37] Shah M.R., Kriedt C.L., Lents N.H., Hoyer M.K., Jamaluddin N., Klein C., Baldassare J. (2009). Direct intra-tumoral injection of zinc-acetate salts tumor growth in a xenograft model of prostate cancer. J. Exp. Clin. Cancer Res..

[38] Costello L.C., Franklin R.B. (2012). Cytotoxic/tumor suppressor role of zinc for the treatment of cancer: An enigma and an opportunity. Expert Rev. Anticancer Ther..

[39] Al-Adham I.S., Gilbert P. (1986). Effect of polyvinylpyrrolidone molecular weight upon the antimicrobial activity of povidone-iodine antiseptics. Int. J. Pharm..

[40] Mosmann T. (1983). Rapid colorimetric assay for cellular growth and survival: Application to proliferation and cytotoxicity assays. J. Immunol. Methods.

[41] Ohno M., Abe T. (1991). Rapid colorimetric assay for the quantification of leukemia inhibitory factor (LIF) and interleukin-6 (IL-6). J. Immunol. Methods.

