# Supplementary material for: Activated Charcoal-Alginate Platform for Simultaneous Local Delivery of Bioactive Agents: At the Nexus of Antimicrobial and Cytotoxic Activity of Zn2+ Ions
Source: Gels. 2024 Nov 8;10(11):724. doi: 10.3390/gels10110724 (PMC11593445; doi:10.3390/gels10110724)
Supplement: Supplementary file 1 [file gels-10-00724-s001.zip › gels-3260623-supplementary.pdf]

gels

Article

Activated Charcoal-Alginate Platform for Simultaneous Local Delivery of Bioactive Agents: At the Nexus of Antimicrobial and Cytotoxic Activity of Zn^{2+} Ions

Andrea Osmokrovic[1,*], Ivan Jancic[2], Zeljko Zizak[3], Marina Milenkovic[2] and Bojana Obradovic[1]

[1] University of Belgrade, Faculty of Technology and Metallurgy, Karnegijeva 4, 11000 Belgrade, Serbia; bojana@tmf.bg.ac.rs
[2] University of Belgrade, Faculty of Pharmacy, Vojvode Stepe 450, 11000 Belgrade, Serbia; ijancic@pharmacy.bg.ac.rs (I.J.); marinama@pharmacy.bg.ac.rs (M.M.)
[3] Institute for Oncology and Radiology of Serbia, Pasterova 14, 11000 Belgrade, Serbia; zizakz@ncrc.ac.rs
* Correspondence: aosmokrovic@tmf.bg.ac.rs; Tel.: +381-11-3370-309; Fax: +381-11-3370-387

Supplementary Materials

Determination of cell survival

Briefly, 20 μl of the MTT dye solution (5 mg/mL of 3-(4,5-dimethylthiazol-2-yl)-2,5-diphenyltetrazolium bromide in phosphate-buffered saline) was added to each well. Samples were incubated for additional 4 h at 37 °C in a humidified atmosphere of 5% CO_2 (v/v). Afterward, 100 μL of 10 % sodium dodecyl sulfate (SDS) were added in order to extract the insoluble formazan, which is the conversion product of the MTT dye by viable cells. Metabolic activity of viable cells in each well is proportional to the absorbance intensity (A, after subtraction of the absorbance of the blank), which was measured by a microtiter plate reader at 570 nm, 24 h later. To determine the cell survival (S%), absorbance values of investigated samples were divided by the absorbance value of the control (control cells grown only in complete medium) and multiplied by 100. All experiments were performed in triplicates.

Results

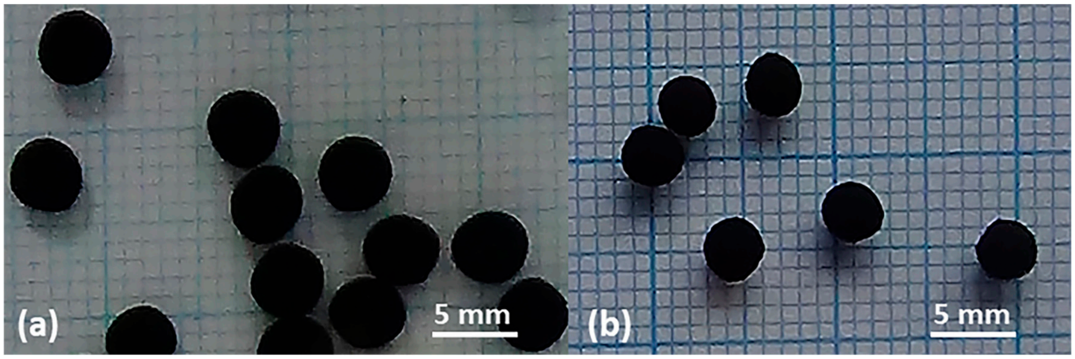

Figure S1. (a) wet ZnA/AC beads; (b) dry ZnA/AC beads (scale bar: 5 mm).

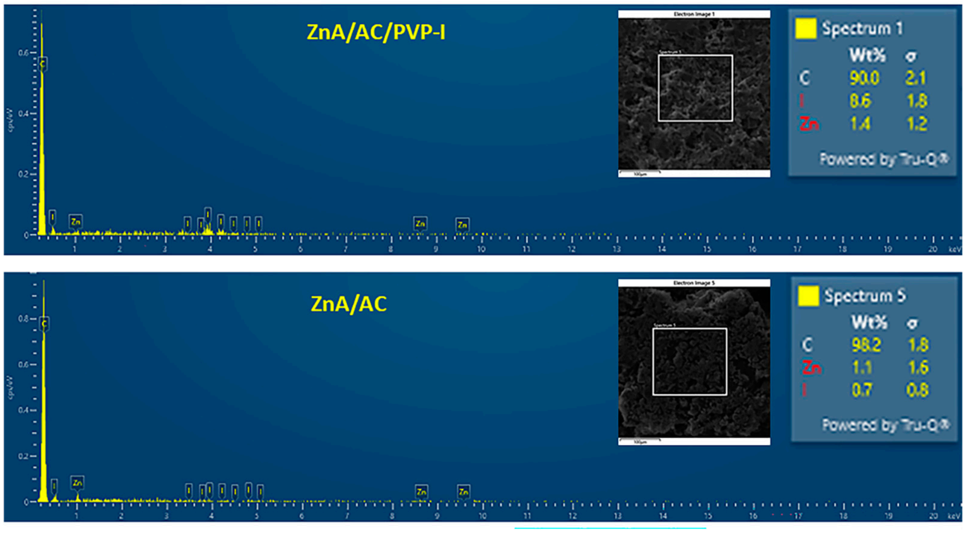

Figure S2. EDX mapping of ZnA/AC/PVP-I and ZnA/AC composite beads.

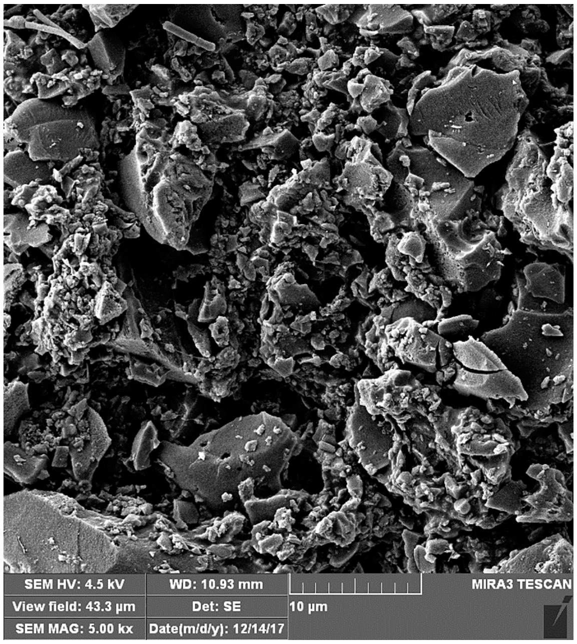

Figure S3. FE-SEM micrograph of cross-section of dry ZnA/AC/PVP-I bead (scale bar: 10 μm).

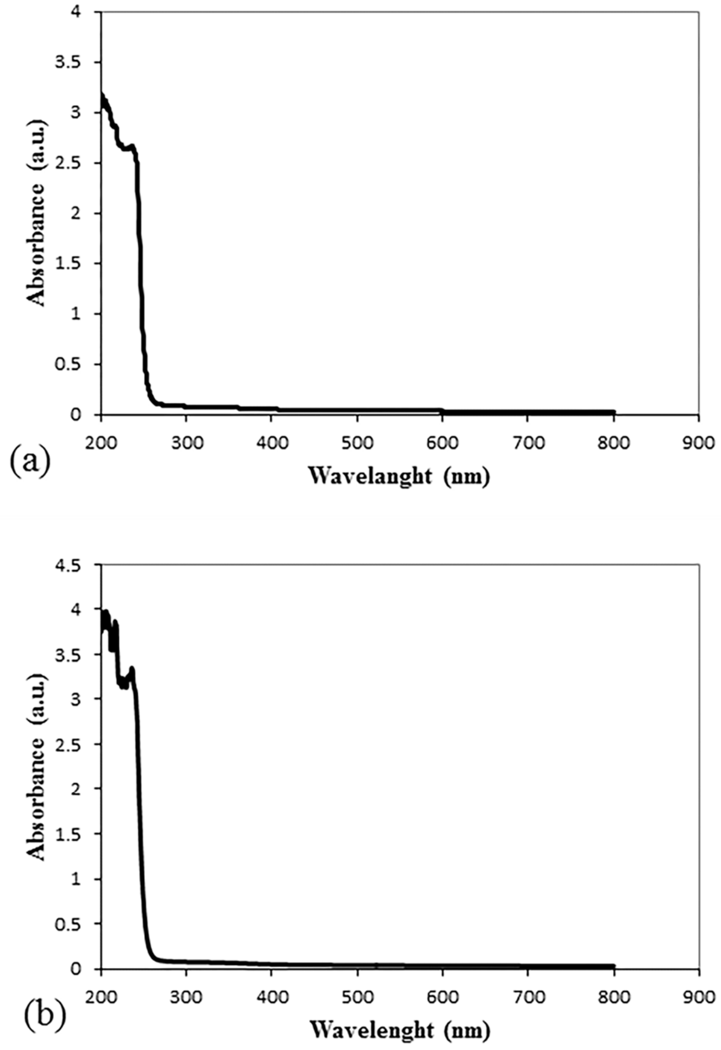

Figure S4. UV-Vis spectra of the :(a) saline solution; (b) dissolved ZnA/AC/PVP-I beads after 48 h in physiological saline solution.
